# Home-Based Long-Term Physical Endurance and Inspiratory Muscle Training for Children and Adults With Fontan Circulation—Initial Results From a Prospective Study

**DOI:** 10.3389/fcvm.2021.784648

**Published:** 2022-02-07

**Authors:** Stefan Dirks, Peter Kramer, Anastasia Schleiger, Hans-Martin Speck, Bernd Wolfarth, Thomas Thouet, Felix Berger, Hannes Sallmon, Stanislav Ovroutski

**Affiliations:** ^1^Department of Congenital Heart Disease—Pediatric Cardiology, Deutsches Herzzentrum Berlin, Berlin, Germany; ^2^Department of Sports Medicine, Charité—Universitätsmedizin Berlin, Berlin, Germany; ^3^Department of Pediatric Cardiology, Charité—Universitätsmedizin Berlin, Berlin, Germany; ^4^German Centre for Cardiovascular Research (DZHK), Berlin, Germany

**Keywords:** Fontan circulation, cardiopulmonary exercise testing (CPET), endurance training, inspiratory muscle training (IMT), quality of life

## Abstract

**Background:**

Patients with congenital heart disease (CHD)—including those after Fontan operation—are encouraged to be physically active.

**Aim:**

To prospectively determine the effects of an individually adapted, home-based cycle ergometer endurance training in combination with inspiratory muscle training (IMT) in pediatric and adult Fontan patients. We, herein, report the results of the initial 10-months follow-up (phase 1).

**Methods:**

18 patients (median age 16.5 years; range 10-43 years) completed baseline check-ups, and 4 and 10 months follow-up visits, which each included cardiopulmonary exercise testing (CPET), bodyplethysmography (including measurement of respiratory muscle strength), and a quality of life questionnaire (PedsQL™). The training program consisted of a home-based cycle ergometer endurance training on a “Magbike® AM-5i/3i” (DKN Technology®, Clermont-Ferrand, France) and IMT with a handheld “POWERbreathe® Medic plus” device. Patients performed 90 min of endurance training per week in addition to IMT (30 breaths per day, 6-7 times per week). After the first 4 months, patients underwent additional interval training.

**Results:**

After 10 months of training, we observed significant increases in maximum relative workload (W/kg, *p* = 0.003) and in maximum inspiratory (MIP, *p* = 0.002) and expiratory (MEP, *p* = 0.008) pressures. Peak VO_2_ values did not increase significantly as compared to baseline (*p* = 0.12) in the entire cohort (*n* = 18), but reached statistical significance in a subgroup analysis of teenage/adult patients (*n* = 14; *p* = 0.03). Patients' subjective quality of life did not show any significant changes after 10 months of training.

**Discussion:**

In Fontan patients, an individually adapted home-based training is safe and associated with improvements in some CPET variables. However, these improvements did not translate into an improved QoL after 10 months. With an unclear, but most likely negative, impact of the COVID-19 pandemic, improvements in QoL may become evident during further follow-up (phase 2 of the study).

## Introduction

The development of the Fontan operation and its subsequent modifications represent a mainstay of surgical palliation in children with single-ventricle malformations (1). Due to continuous refinements in surgical techniques, perioperative care, and medical and interventional treatment strategies, which have resulted in improvements in early and late mortality, a growing number of affected children enter into adolescence and adulthood (2–4). Nonetheless, the basic hemodynamic limitations of the Fontan circulation and major long-term comorbidities have remained vastly unchanged (4–6).

Specifically, increased pulmonary vascular resistance and a non-pulsatile pulmonary flow pattern may lead to systemic venous congestion and reduced ventricular preload, thereby decreasing efficacy of the Frank-Starling mechanism. In addition, a decreased heart rate reserve, ventricular fibrosis and/or hypertrophy, and an impaired systolic and/or diastolic function all result in a reduced capacity to increase stroke volume and cardiac output during physical activity (2, 5). Furthermore, Fontan patients exhibit a restrictive lung function pattern, an impaired pulmonary diffusing capacity, and a reduced maximal oxygen uptake (7), which, in turn, are associated with decreases in health-related quality of life (8–11) and exercise tolerance (12–14).

Despite these intrinsic limitations, short term exercise programs and endurance training have been shown to positively effect stroke volume, cardiac output, and lung function, thereby increasing overall physical function, exercise capacity, and quality of life (15–18). Furthermore, inspiratory muscle training (IMT) has been shown to improve cardiac output (19). Thus, patients with congenital heart disease (CHD)—including those after Fontan operation—are encouraged to be physically active and exercise training is recommended on an individual basis (20).

Recently, CHD sports programs were designed to specifically address training and medical needs of CHD patients in several countries. However, a widespread geographic distribution of the Fontan population may limit access to those programs. Therefore, home-based programs have been introduced which demonstrated a similar efficacy as compared to hospital-based programs without an increasing rate of adverse events (17, 21–26). These results led to the recent recommendation to integrate home-based exercise training programs in the follow-up care of patients undergoing Fontan surgery (17, 23). However, efficacy results of individually adapted, home-based training protocols specifically designed for Fontan patients are still sparse. Therefore, we aimed to prospectively determine the effects of an individually adapted, home-based cycle ergometer endurance training (including interval training) in combination with IMT in a cohort of pediatric and adult Fontan patients. Herein, we report the initial results of a 10-months follow-up.

## Methods

### Study Design and Population

We conducted a single center prospective study on the long-term impact of a home-based training with individually prescribed and adapted endurance training in combination with inspiratory muscle training (IMT). The endurance training protocol (see below) was developed in co-operation with the Department of Sports Medicine at Charité—Universitätsmedizin Berlin. Patients were enrolled between March 2018 to March 2021. The study is divided into two study periods, with follow-up examinations in phase 1 taking place after 4 and 10 months, while follow-up visits in phase two are planned after 16 and 22 months of training. Herein, we report the results from phase 1.

Participants with Fontan circulation had to be at least 6 years of age. Informed consent of patients or legal guardians was obtained prior to enrollment. The study was reviewed and approved by the Institutional Review Board of Charité—Universitätsmedizin Berlin (EA2/244/17). The patients' medical history and routine cardiac examination were reviewed, and upon inclusion patients underwent a baseline check-up which encompassed cardiopulmonary exercise testing (CPET), bodyplethysmography, including measurement of respiratory muscle strength (CASETM ES Version 6.73, GE Health Care, Germany) (27) and a standardized health-related quality of life questionnaire (PedsQL™–Pediatric Quality of Life Inventory)—including the cardiac module (28). Within the PedsQL™, different questions are allocated to several sub-categories (psychosocial, physical health, heart problems and treatment, treatment anxiety, cognitive problems, and communication). Childrens' body weight was evaluated using the reference values of the KiGGS' data (29). Lung function was evaluated using the GLI reference values (30, 31). Blood pressure values were evaluated according to the recommendations of the National High Blood Pressure Education Program Working Group on High Blood Pressure in Children and Adolescents (32).

### Training Protocol

The training program consisted of a home-based cycle ergometer endurance training on a “Magbike® AM-5i/3i” (DKN Technology®, Clermont-Ferrand, France). Training was adapted individually to each patient's pre-training and check-up values. Patients performed 90 min of training per week, in 3 or 6 sessions of 30 or 15 min at a time, respectively (Table 1). Workload was set in 5 watts steps to 55% of maximum workload obtained during the latest check-up, respectively. After the first follow-up visit (4 months), patients underwent additional interval training (four intervals of 4 min at 80% of the maximum workload separated by 4 min of rest). For safety reasons, patients were urged to perform their training only when a person, who could assist or send for help in case of medical problems, was present. Prior to the initiation of home-based training, all patients underwent a baseline check-up, including CPET evaluation. If no adverse events, including symptomatic hyotonia, syncope, severe desaturation or arrhythmias occurred, patients were eligible for home-based training. An individual safety heart rate limit was set for each patient (blood pressure and saturation monitoring was not available at home in most patients). In addition to endurance training, the study participants executed an inspiratory muscle training with a handheld “POWERbreathe® Medic plus” device (POWERbreathe®, Winsen, Germany) with adjustable expiratory resistance. Inspiratory muscle training was performed at least 6 times a week for 30 consecutive slow and deep breaths. The effort was to be assessed on the 6-20 Borg scale. Participants were to adjust the resistance to achieve a value of 12-15 on the Borg scale.

**Table 1:** Study protocol.

**Baseline**		
	CPET + Body + MIP/MEP + QoL	
**Training period 1**		
16 weeks (4 months)	Inspiratory muscle training (IMT)	30-50% of MIP 1 x 30 breaths per day (6-7 x per week)
	Ergometry	55% of W at VO2max at baseline 3 x 30 min per weeks or 6 x 15 min
	Training diary	Monthly check + weekly calls
**Follow-up 1**		
16 weeks (4 months)	CPET + Body + MIP/MEP + QoL	
**Training period 2**		
24 weeks (10 months)	Inspiratory muscle training (IMT)	1 x 30 breaths per day with adapted pressure (6-7 x per week)
	Ergometry	Adapt: 55% of W at VO2max at follow-up 1 2 x 45 min per weeks or 5 x 18 min + Interval training (4 x 4 min at 80% W at VO2max with 3 min of recovery each)
	Training diary	Monthly check + weekly calls
**Follow-up 2**		
24 weeks (10 months)	CPET + Body + MIP/MEP + QoL	

*Body, bodyplethysmography; CPET, cardiopulmonary exercise testing; MEP, Maximum expiratory pressure; MIP, maximum inspiratory pressure*.

Monitoring of the training included a training diary, filled out by the participants or their parents, which was checked on a monthly basis. Regular phone calls complemented the assessments of compliance and supported motivation. Based on the interviews, compliance was excellent among all 18 patients who completed the study (completion of at least 85% of the prescribed exercises). Of note, 7/25 initially enrolled patients had to be excluded before final analyses due to a low compliance or voluntary termination of the study.

### Statistics

Statistical analysis was carried out using SPSS Version 25 (SPSS Inc., Chicago, IL, USA) In all analyses at least one variable was not in normal distribution, therefore non-parametric tests were used. Differences between groups (the three time points) were analyzed by the non-parametric Friedman test for three or more paired samples. *P*-values <0.05 were considered statistically significant. In addition, a-priori power analysis was performed to estimate the required sample size (based on anticipated changes in the PedsQL scores): Assuming a sample size of *n* = 20, significant changes can be seen with a minimal effect size of 0.66 (power 80%, alpha-level 0.05), which would be equivalent to, e.g., an increase of 5% (standard deviation 8%). With only *n* = 10 children enrolled, the required effect size would have to be 0.95 (power 80%, alpha-level 0.05), e.g., equivalent to an increase of 12% (standard deviation 13%).

## Results

### Demographics

A total of 25 patients were initially enrolled in the study, out of which 18 patients (median age 16.5 years; range 10-43 years) were included in the final analyses. Seven out of the 25 initially enrolled patients were excluded due to a low compliance or voluntary termination of the study. Compliance was assessed by means of a training diary and weekly telephone interviews with patients and parents. These calls were also supposed to work as a motivational strategy. 11/18 (61.1%) were male and 7/18 (38.9%) female. Baseline body weight in adults ranged between 37 and 84 kg (median 65 kg), while in children weight ranged between the 5th and 79th percentile (median 35th percentile). Details on the underlying congenital cardiac malformation are provided in Table 2. Of note, three patients (16.7 %) had evidence of an open fenestration at the latest echocardiography before initiation of training and two patients (11.1%) had an epicardiac pacemaker. Cardiac medication remained stable during the study period in all 18 patients. The median oxygen saturation at rest was 97% (range 89-100%). At baseline, the mean resting systolic and diastolic blood pressures in adults were 121 (range 69-143) mmHg and 79 (range 50-86) mmHg. In children, the mean resting systolic blood pressures at baseline was at the 71st (range 4th to 99th) percentile. 13/18 patients underwent cardiac catheterization during the 5 years preceding baseline (median 8 months). Invasively obtained mean pulmonary artery pressures (mPAP) ranged between 7 and 14 mmHg (median 12 mmHg), indicating stable Fontan circulation (Table 2). The remaining patients showed no indication for invasive diagnostics due to reassuring general condition, echocardiographic results and high oxygen saturations (33).

**Table 2:** Baseline patients' characteristics.

	**Age**	**Gender**	**Height (cm)**	**Weight (kg) baseline/4 months/10 months**	**Age at Fontan completion**	**Cardiac malformation**	**Comment**	**Medication**	**Mean pulmonary artery pressure [mmHg]**
01	22	F	169	62/55/55	2	TA, PA, intact VS	Fenestration	VKA	14
02	13	F	153	40/43/45	4	PA, VSD, TGA	-	ACEI, VKA	11
03	15	F	170	50/50/51	3	TA, VSD, pulmonary artery ligation	-	VKA	11
04	12	M	158	40/42/45	3	TA, TGA, VSD, s/p Damus-Kaye-Stansel operation	-	VKA	-
05	43	M	167	69/70/70	17	TA, PA, VSD, occlusion left subclavian artery	-	ACEI, HCT, MCRA, VKA, Propafenone, Ivabradine	-
06	12	M	148	49/51/54	3	TA, VSD, sick sinus syndrome	DDD-Pacemaker	ACEI, PDE5 inhibitor, VKA	14
07	20	M	185	68/67/67	5	TA, VSD, coarctation of the aorta, DORV, s/p Damus-Kaye-Stansel operation	-	VKA	13
08	24	M	190	71/70/70	3	TA	-	ASA, HCT	14
09	26	M	187	84/86/88	6	TA	-	VKA	12
10	24	F	156	37/38/38	3	DILV, sick sinus syndrome, AV Block II°	-	ACEI, VKA	11
11	12	M	143	46/47/49	3	HLHS, coarctation of the aorta	Fenestration	ACEI, VKA	-
12	11	M	143	29/31/31	4	DORV, DILV, PS	-	VKA	-
13	11	M	149	38/40/41	3	MA, DORV, VSD	-	ACEI, HCT, VKA	-
14	11	M	150	46/49/51	3	TA, VSD, PS	DDD-Pacemaker	ACEI, VKA	7
15	10	F	135	32/36/39	9	PA, VSD, tricuspid valve dysplasia	Fenestration	ACEI, PDE5 inhibitor, VKA	11
16	18	F	164	57/60/59	3	TA, PS	-	ASA	8
17	27	F	162	58/59/60	24	DORV,VSD,TGA, RVOT stenosis	Fenestration	HCT, MCRA, VKA	12
18	36	F	158	60/59/60	9	DILV, VSD, PS	-	ACEI, ASA	13

*DILV, double inlet left ventricle; DORV, double outlet right ventricle; F, female; HLHS, hypoplastic left heart syndrome; M, male; MA, mitral atresia; PA, pulmonary atresia; PS, pulmonary stenosis; RVOT, right ventricular outflow tract; TA, tricuspid atresia; TGA, transposition of great arteries; VS, ventricular septum; VSD, ventricular septal defect; ACEI, ACE inhibitors; ASA, acetylsalicylic acid; HCT, hydrochlorothiazide; MCRA, mineralocorticoid receptor antagonist; VKA, vitamin K antagonist*.

### Cardiopulmonary Function

**Figure 1:**
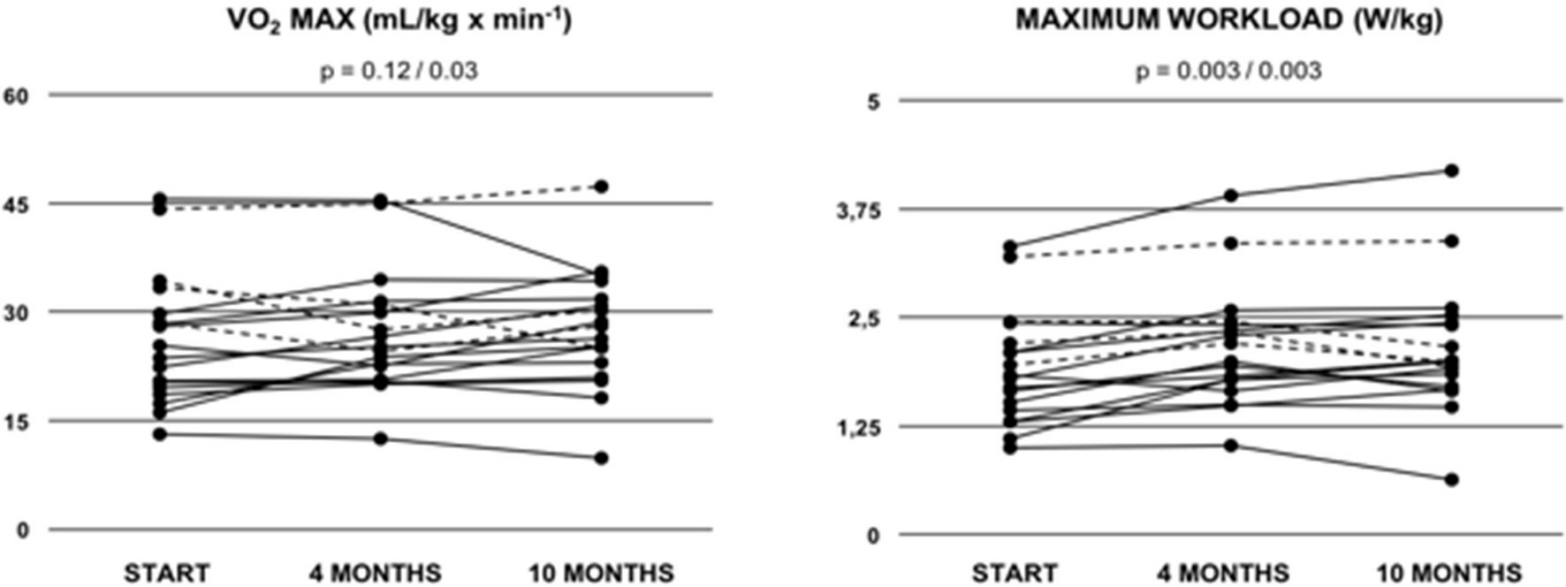
Peak oxygen uptake (VO_2_ MAX) and maximum relative workload (W/kg) in Fontan patients during the 10-months training period. Values are given at baseline (start), and after 4 and after 10 months of combined endurance and inspiratory muscle training. Each line represents one patient. Full lines represent teenage/adult patients, while dashed lines indicate patients who remained younger than 13 years throughout the study period. The first *p*-value refers to the total cohort of 18 patients, while the second *p*-value refers to the teenage/adult subgroup analyses only (*n* = 14).

Cardiopulmonary function was evaluated at baseline, and after 4 and 10 months of training (Table 3). After 10 months of follow-up, peak VO_2_ values did not increase significantly as compared to baseline (mean 26.50 ± 2.10 ml/min/kg to 28.10 ± 1.95 ml/min/kg; *p* = 0.12) (Figure 1). Of note, in the sub-cohort of teenage/adult patients (patients who turned 13 or older during the study period, *n* = 14), the increase in peak VO_2_ reached statistical significance after 10 months of training (23.50 ± 3.86 ml/min/kg to 26.09 ± 2.00 ml/min/kg; *p* = 0.03, Figure 1; Table 4). Slope values did not decrease significantly during the 10-months training period, in both the overall cohort (*p* = 0.07, Table 3) and the sub-cohort of teenage/adult patients (*p* = 0.09, Table 4). In addition, in the total cohort maximum relative workload increased within 10 months of training from a mean of 1.92 ± 0.15 W/kg to 2.17 ± 0.19 W/kg (*p* = 0.03) (Table 3; Figure 1).

**Table 3:** Cardiopulmonary exercise testing (CPET, all patients, *n* = 18).

	**Baseline**	**4 months**	**10 months**	***P***
HF (rest) [bpm]	91.50 ± 3.28	91.44 ± 3.85	85.41 ± 3.33	0.09
HF (max) [bpm]	158.67 ± 7.56	165.22 ± 6.39	161.59 ± 7.63	0.12
Heart rate reserve [bpm]	67.17 ± 6.93	73.78 ± 5.50	76.18 ± 6.16	0.058
Max. power/weight [W/kg]	1.92 ± 0.15	2.14 ± 0.16	2.17 ± 0.19	**0.003**
VO_2_max [ml/min/kg ]	26.50 ± 2.10	26.99 ± 1.98	28.10 ± 1.95	0.12
AT/reference [%]	48.88 ± 4.08	47.88 ± 3.51	52.00 ± 4.06	0.12
VE/VCO_2_ slope	33.02 ± 1.32	33.42 ± 1.10	32.51 ± 1.45	0.07
TCS (rest) [%]	96.18 ± 0.82	95.53 ± 0.97	95.94 ± 0.63	0.98
TCS (max. capacity) [%]	92.67 ± 1.07	92.44 ± 1.15	92.00 ± 1.18	0.29
VC_insp_/reference [%]	77.75 ± 5.05	81.21 ± 4.78	81.55 ± 4.64	0.87
TLC/reference [%]	85.64 ± 3.64	85.61 ± 3.82	85.28 ± 4.42	0.98
FEV_1_/VC_insp_ [%]	92.83 ± 1.95	92.76 ± 1.66	90.91 ± 2.33	0.16
MEP [kPa]	6.87 ± 0.80	7.71 ± 0.84	8.17 ± 1.00	**0.008**
MIP [kPa]	6.41 ± 0.63	8.90 ± 0.89	8.95 ± 0.97	**0.002**

*Baseline results and results after 4 and 10 months of training are shown with corresponding P-values (non-parametric Friedman test for three or more paired samples). Significant values are shown in bold. All CPETs were maximal test (RER > 1.0). HF, heart frequency; AT, anaerobic threshold; TCS, transcutan oxygen saturation; FEV1, forced expiratory pressure in 1 s; VCinsp, inspiratory vital capacity; MEP, maximal expiratory pressure; MIP, maximal inspiratory pressure; TLC, total lung capacity*.

**Table 4:** Cardiopulmonary exercise testing (CPET, teenage/adult subgroup, *n* = 14).

	**Baseline**	**4 months**	**10 months**	***P***
HF (rest) [bpm]	89.29 ± 4.08	90.36 ± 4.88	83.14 ± 3.71	0.23
HF (max) [bpm]	155.21 ± 8.76	162.21 ± 7.62	157.79 ± 8.51	0.11
Heart rate reserve [bpm]	65.93 ± 7.78	71.86 ± 6.24	74.64 ± 6.71	0.085
Max.power/weight [W/kg]	1.75 ± 0.15	2.00 ± 0.15	2.02 ± 0.17	**0.003**
VO_2_max [ml/min/kg ]	23.50 ± 3.86	25.43 ± 2.15	26.09 ± 2.00	**0.03**
AT/reference [%]	42.46 ± 2.94	44.94 ± 3.69	48.07 ± 3.38	0.09
VE/VCO_2_ slope	32.17 ± 1.71	33.14 ± 1.42	31.31 ± 1.61	0.09
TCS (rest) [%]	95.92 ± 0.98	95.36 ± 1.10	95.92 ± 0.67	0.97
TCS (max. capacity) [%]	92.43 ± 1.31	91.93 ± 1.33	92.31 ± 1.42	0.42
VC_insp_/reference [%]	71.15 ± 5.12	70.21 ± 4.94	69.00 ± 5.50	0.56
TLC/reference [%]	90.48 ± 2.26	91.13 ± 1.96	89.70 ± 3.00	0.61
FEV_1_/VC_insp_ [%]	92.23 ± 4.78	96.14 ± 5.12	103.64 ± 4.11	0.09
MEP [kPa]	6.25 ± 0.80	8.84 ± 1.08	7.87 ± 1.18	**0.045**
MIP [kPa]	6.63 ± 0.73	9.35 ± 1.04	8.38 ± 1.07	**0.014**

*Baseline results and results after 4 and 10 months of training are shown with corresponding p-values (non-parametric Friedman test for three or more paired samples). Significant values are shown in bold. All CPETs were maximal test (RER > 1.0). HF, heart frequency; AT, anaerobic threshold; TCS, oxygen saturation; FEV1, forced expiratory pressure in 1 s; VCinsp, inspiratory vital capacity; MEP, maximal expiratory pressure; MIP, maximal inspiratory pressure; TLC, total lung capacity*.

Furthermore, in both cohorts no significant changes in resting heart rate or functional heart rate reserve could be demonstrated (Table 3). Of note, at baseline, there was no significant difference in the drop of oxygen saturation during exercise when comparing patients with and those without fenestration (*U* = 19.5, Z = −0.748, *p* = 0.477). Consistently, these results did not change after 10 months of training (*U* = 12.0, Z = −1.022, *p* = 0.307).

### Pulmonary Function

**Figure 2:**
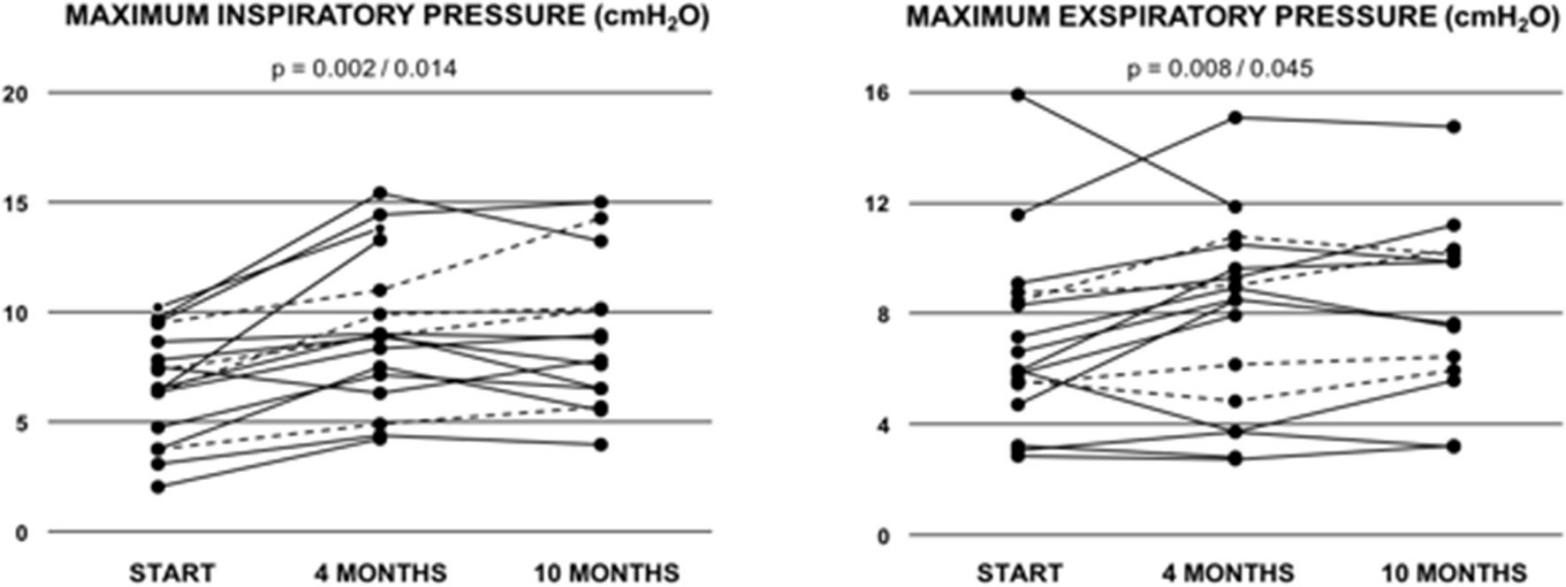
Maximum inspiratory pressure (MIP) and maximum exspiratory pressure (MIP) in Fontan patients during the 10-months training period. Values are given at baseline (start), and after 4 and after 10 months of combined endurance and inspiratory muscle training. Each line represents one patient. Full lines represent teenage/adult patients, while dashed lines indicate patients who remained younger than 13 years throughout the study period. The first *p*-value refers to the total cohort of 18 patients, while the second *p*-value refers to the teenage/adult subgroup analyses only (*n* = 14).

9/18 (53%) showed normal pulmonary function, while 6/18 (35%) exhibited a restrictive and 2/18 (12%) an obstructive pattern at baseline. One patient did not tolerate bodyplethysmography at baseline. As expected, vital capacity (VC), total lung capacity (TLC) and FEV1/VC did not show any significant improvements after 10 months of training. However, in the cohort of patients from all age groups maximal inspiratory pressure significantly increased from 6.41 ± 0.63 to 8.95 ± 0.97 kPa (*p* = 0.002; Table 3; Figure 2) and maximal expiratory pressure from 6.87 ± 0.80 to 8.17 ± to 1.00 kPa (*p* = 0.008, Table 3; Figure 2). Again, similar results were obtained from the sub-cohort analysis of teenage/adult patients (Table 4)

### Quality of Life

Patients' subjective quality of life as determined by the PedsQL^TM^ questionnaires did not show any significant changes after 10 months of training (in neither of the sub-categories -psychosocial, physical health, heart problems and treatment, treatment anxiety, cognitive problems and communication, data not shown).

## Discussion

We herein show, that an individually adapted home-based training is safe and associated with improvements in some CPET variables. However, these improvements did not translate into an improved QoL after 10 months. Importantly, none of the patients experienced any adverse events during home-based training such as syncope, symptomatic arrhythmia or acute hospitalization.

During the last decades, survival rates in children with CHD have increased substantially. Currently, more than 97% of children with CHD can be expected to reach adulthood (2, 34–36). With an improved long-term survival, aspects of long-term morbidity, cardiopulmonary capacity, pulmonary function, reproductive and psychosocial health and quality of life require closer attention. In patients with Fontan circulation, exercise capacity has been reported to be reduced to about 50-60% (10, 13). However, impaired exercise capacity is known to be associated with adverse outcome in CDH patients, including Fontan patients. Furthermore, CPET is indicated in Fontan patients with pulmonary vascular disease (37) and should be performed on a regular basis in all Fontan patients if possible (38).

Paridon et al. observed peak VO_2_ values within the normal range in only 28% of patients. However, a majority of individuals (63%) exhibited an anaerobic threshold within the normal range, suggesting that many Fontan patients tolerate a high level of submaximal and non-maximal activity (3). Thus, endurance training at the lower end of the individually tolerated workload (defined as W/kg at VO_2_max = 100%) seems adequate for Fontan patients. However, long-term data on training studies is still sparse in the Fontan population. A recent review article (39) showed that out of 245 individuals reported in the literature, only 88 (36%) patients trained for more than 3 months and only 30 individuals (12%) trained in studies lasting longer than 8 months. We, herein, report on the 10-months follow-up of 18 patients who underwent an individually adapted, home-based ergometer endurance training (including interval training) in combination with IMT.

### Cardiopulmonary Impact

Exercise intolerance in Fontan patients is caused by multiple factors, which include the lack of a subpulmonary ventricle (pump function), chronotropic incompetence, restrictive lung function, reduced muscle mass, and general deconditioning, among others (40, 41). While healthy people improve their cardiac output by increasing both heart rate and stroke volume during exercise, in the Fontan circulation, increases in heart rate are crucial to enhance cardiopulmonary exercise capacity due to a reduced capacity to increase preload (lack of a subpulmonary ventricle) (42). In addition, heart rate reserve is further reduced in several patients by medication and/or iatrogenic damage of the sinus node (43). After 10 months of training, we did not see significant reductions in resting heart rate or increases in functional heart rate reserve (Tables 3, 4). Due to the limited size of our total cohort, a meaningful sub-cohort analysis of patients on specific antiarrhythmic medications and/or sick sinus syndrome was not feasible. However, heart rate reserve between patients with and those without pacemaker did not differ significantly (*p* = 0.9). This was also true when all patients with impaired chronotopy (antiarrhythmic medication with betablockers, ivabradin or pacemaker patients) were compared to patients without pacemakers or antiarrhythmic medication (data not shown). Thus, it remains unclear if the lack of significant improvements in functional heart rate reserve after training represents an intrinsic limitation in all Fontan patients or if medications and specific heart rhythm anomalies are responsible for these results. Furthermore, it is likely that subtle improvements may only become evident during a longer training period.

Besides heart rate reserve, peak VO_2_ and VE/VCO_2_ slope are major indicators of cardiopulmonary exercise capacity. Healthy people reach their maximum of peak VO_2_ at early adulthood, with values beginning to drop around the age of 30 years. However, in Fontan patients peak VO_2_ values already start to decrease disproportionally early during adolescence (10, 44). In our teenage/adult cohort we were able to demonstrate significant improvements in peak VO_2_ after 10 months of training, while we did not see significant improvements in the total cohort of 18 patients. In their recent review article incorporating 16 studies on endurance training in Fontan patients, Scheffers et al. report that only 9 out of 16 reviewed studies demonstrated improvements in peak VO_2_ (39). Differences in results among studies are likely caused, at least in part, by different follow-up durations as functional parameters, such as peak VO_2_ and slope, usually require some time of training before significant improvements become evident.

Of note, previous studies demonstrated significantly lower oxygen saturation values at rest (45) and a pronounced systemic desaturation during exercise in patients with fenestration as compared to those without fenestration (46). In the cohort presented herein, we were unable to confirm these results as our results did not show any differences in saturation values between groups. However, our study only included four patients with fenestration precluding a statistically meaningful subgroup analysis. In addition, the presence of fenestrations was documented by echocardiography preceding the baseline evaluation only. Thus, their hemodynamic relevance remains unclear in our patients. Of note, we did not observe any significant differences (CPET, pulmonary function, QoL) between the patients with and those without fenestration (data not shown).

### Pulmonary Function

About one third of the patients in our cohort exhibited a restrictive breathing pattern at baseline, in line with previous reports on lung function test results in Fontan patients (7, 47). The observed restrictive breathing pattern is likely caused by multiple factors, which include diaphragmatic paralysis (48, 49) and a reduced thoracic mobility due to repeated thoracotomies with presence of less flexible scar tissue (49). Previous results on the impact of different physical training programs on lung function testing in Fontan patients revealed conflicting results. While, similar to our results, Fritz et al. failed to show significant improvements in vital capacity, total lung capacity and Tiffeneau-Index (FEV1/VC) after daily inspiratory muscle training over 10 months (50), Hedlund et al. demonstrated an increased vital capacity after endurance training (7).

We observed significant increases in maximum power/weight ratio, as well as in maximum inspiratory (MIP) and expiratory (MEP) pressures after 10 months of combined endurance and inspiratory muscle training. MIP and MEP values at baseline ranged between the 3rd and 25th percentile (P3-P25) and increased to P10-P50 during training (51). Laohachai et al. (19) previously reported that improvements in MIP were associated with positive effects on cardiac output in Fontan patients after 6-week of IMT. Thus, one might speculate that higher inspiratory muscle strength may increase pulmonary blood flow (by improving the “thoracic pump”) and subsequently improve preload, cardiac output and exercise capacity (19). Furthermore, the combination of different training modalities, such as IMT and endurance training may, at least theoretically, booster the positive effects of training in Fontan patients as an improved “thoracic pump” and an overall improved cardiorespiratory capacity both enhance overall physical fitness at multiple circulatory target sites.

### Quality of Life

Although we did observe improvements in several parameters of cardiopulmonary exercise capacity, those changes did not translate into patients' subjective quality of life as determined by the PedsQL questionnaires after 10 months of training. Previously, several authors found improvements in quality of life indices by training interventions in Fontan patients (11, 18, 28, 37, 52), while others reported unchanged QoL (23). Although several factors, such as differences in training protocols, duration and compliance, might contribute to these discrepancies in previously reported results, self-reported QoL is generally influenced by a variety of possible confounders (parenteral influence, bias by peer comparison, etc.). In addition, some categories of the quality-of-life questionnaire, such as “treatment anxiety” and “cognitive problems and communication” are unlikely to change by physical training programs.

Furthermore, a home-based training, as performed here, almost completely lacks the social benefit of training together. However, home-based protocols may enable an easy individual adaptation of training plans and facilitate inclusion of more patients as compared to a standard group training at a certain location. While we believe that a longer follow-up may be required to detect improvements in QoL, the study was conducted during a time heavily affected by the COVID-19 pandemic with its detrimental effects on families, social life, school, work, and activities, which likely had a negative impact on general QoL. In this regard, several patients affected by CHD and especially older Fontan patients reduced many of their social activities in order to decrease their risk of infection. Of note, the PedsQL questionnaire had been initially designed for patients up to 18 years. However, in order to allow for comparative analyses we used PedsQL in all patients (children, teenagers and adults). Furthermore, adult patients with Fontan circulation often face specific challenges, which are similar to their situation during childhood (high dependence on parental support etc.).

### Limitations

In addition to the previously mentioned confounders, such as the co-occurrence of the COVID-19 pandemic, further limitations apply when interpreting the results presented in this study. First, the number of patients is limited and patients differed in regard to underlying age, gender, cardiac defect, surgical modifications, and medical treatment. Nevertheless, compared to previously reported studies on physical training in Fontan patients, our cohort is of comparably large size and followed-up for a relatively long period of time (10 months during phase 1). In addition, differences in motivation, other health-related problems, and differences in socio-economic backgrounds, supervision by parents, self-awareness of benefit, cognitive abilities and technical execution of training represent further confounders that were unaccounted for in this analysis (27). In addition, due to learning effects, lung function tests are known to improve over time, especially in patients that perform lung function tests for the first time. While we cannot rule out the possibility that learning effects might have contributed to the results, CPET testing and assessment of inspiratory muscle strength is routinely performed during follow-up for all Fontan patients and we usually do not see improvements over time. However, the well-known effects of learning cannot firmly be ruled out. Furthermore, Fontan patients are especially prone to malnutrition and often exhibit a body composition with low muscle mass. Thus, the combined effect of nutritional management together with different physical training programs needs to be evaluated in further studies.

## Conclusions

In conclusion, we show that individually adapted aerobic endurance training in combination with IMT is safe and associated with improvements in certain CPET variables in Fontan patients in a home-based setting. While some CPET measures improved, these improvements did not (yet) translate into an improved QoL. With an unclear, but most likely negative, impact of the COVID-19 pandemic, improvements in QoL may be expected during further follow-up (phase 2 of the study). Additional research efforts on the long-term role of individualized training programs are warranted in order to facilitate improvements (or to prevent premature deterioration) of physical endurance and QoL in Fontan patients.

## Data Availability Statement

The raw data supporting the conclusions of this article will be made available by the authors, without undue reservation.

## Ethics Statement

The studies involving human participants were reviewed and approved by Charité Institutional Review Board (EA2/244/17). Written informed consent to participate in this study was provided by the participants' legal guardian/next of kin.

## Author Contributions

SD, PK, HS, and SO planned and conducted the study. SD and H-MS wrote the manuscript. All authors contributed to data collection, analysis, interpretation, and approved the final version of the manuscript.

## Funding

We acknowledge support from Stiftung KinderHerz (Germany).

## Conflict of Interest

The authors declare that the research was conducted in the absence of any commercial or financial relationships that could be construed as a potential conflict of interest.

## Publisher's Note

All claims expressed in this article are solely those of the authors and do not necessarily represent those of their affiliated organizations, or those of the publisher, the editors and the reviewers. Any product that may be evaluated in this article, or claim that may be made by its manufacturer, is not guaranteed or endorsed by the publisher.
